# The design, fate and impact of a hospital-wide training program in evidence-based medicine for physicians – an observational study

**DOI:** 10.1186/s12909-016-0601-9

**Published:** 2016-03-08

**Authors:** Johan Thor, Daniel Olsson, Jörgen Nordenström

**Affiliations:** The Jönköping Academy for Improvement of Health and Welfare, Jönköping University, P O Box 1026, SE-551 11 Jönköping, Sweden; The Medical Management Centre, Department for Learning, Informatics, Management and Ethics, Karolinska Institutet, Tomtebodavägen 18A, 5th floor, SE-171 77 Stockholm, Sweden; Unit of Biostatistics, Department of Epidemiology, Institute for Environmental Medicine (IMM), Karolinska Institutet, Nobels väg 13, SE-171 77 Stockholm, Sweden; Department of Molecular Medicine and Surgery (MMK), Karolinska Institutet, K1 Karolinska University Hospital, Solna (L1:00), SE-171 76 Stockholm, Sweden

**Keywords:** MeSH: Delivery of Health Care; Integrated/organization & administration*, Education, Medical; Evidence-Based Medicine/education*, Inservice Training/organization & administration*, Problem-Based Learning/methods*, Program Development; Program Evaluation

## Abstract

**Background:**

Many doctors fail to practice Evidence-Based Medicine (EBM) effectively, in part due to insufficient training. We report on the design, fate and impact of a short learner-centered EBM train-the-trainer program aimed at all 2400 doctors at the Karolinska University Hospital in Sweden on the heels of a tumultuous merger, focusing particularly on whether it affected the doctors’ knowledge, attitudes and skills regarding EBM.

**Methods:**

We used a validated EBM instrument in a before-and-after design to assess the impact of the training. Changes in responses were analyzed at the individual level using the Wilcoxon matched pairs test. We also reviewed documentation from the program – including the modular EBM training schedule and the template for participants’ Critically Appraised Topic reports – to describe the training’s content, design, conduct, and fate.

**Results:**

The training, designed to be delivered in modules of 45 min totaling 1.5 days, failed to reach most doctors at the hospital, due to cost cutting pressures and competing demands. Among study participants (*n* = 174), many reported suboptimal EBM knowledge and skills before the training. Respondents’ strategies for solving clinical problems changed after the training: the proportion of respondents reporting to use (or intend to use) secondary sources “Often/very often” changed from 5 % before the training to 76 % after the training; in parallel, reliance on textbooks and on colleagues fell (48 to 23 % and 79 to 65 %, respectively). Participants’ confidence in assessing scientific articles increased and their attitudes toward EBM became more positive. The proportion of correct answers in the EBM knowledge test increased from 52 to 71 %. All these changes were statistically significant at *p* < 0.05.

**Conclusions:**

Many study participants, despite working at a university hospital, lacked basic EBM knowledge and skills and used the scientific literature suboptimally. The kind of short learner-centered EBM training evaluated here brought significant improvements among the minority of hospital doctors who were able to participate and, if applied widely, could contribute to better, safer and more cost-effective care.

**Electronic supplementary material:**

The online version of this article (doi:10.1186/s12909-016-0601-9) contains supplementary material, which is available to authorized users.

## Background

Clinicians face the daunting task of addressing patient needs by drawing on the ever-increasing amount of knowledge yielded by basic science and clinical research. The development of evidence-based medicine (EBM) can be seen as an effort to enhance the application of scientific knowledge to achieve better, safer and more cost-effective care [1]. EBM proponents argue that “Doctors owe it to themselves and their patients to make sure that they keep up with what’s new and important.” [2] Several studies have found, however, that there is a considerable gap between the best evidence and the care actually delivered [3–5].

A number of barriers to EBM practice have been identified, including time constraints, limited access to electronic information resources, poor information-searching skills, lack of motivation and an inhospitable institutional culture [6, 7].

Although EBM has been widely accepted as a cornerstone of good healthcare [8–10], many clinicians lack adequate familiarity with its principles and practices [11–13]. While EBM training has been integrated in medical school curricula, many currently practicing physicians graduated before this development. Furthermore, a systematic review of the relationship between clinical experience and the quality of health care suggests that “physicians who have been in practice for more years and older physicians possess less factual knowledge, are less likely to adhere to appropriate standards of care, and may also have poorer patient outcomes [14].”

In theory, training in EBM should help [15]. Numerous examples of EBM training interventions have been described [16–19] but evidence on the effectiveness of different EBM training designs has been slow to accumulate [20, 21]. The heterogeneity of training programs and the methodological limitations of many evaluative studies have made it difficult to conclude what works best [22–25].

While we already know that EBM is best learned when the training is learner-centered, patient-related, participant-activating, and problem-based [26, 27], it is less clear how best to organize effective EBM training throughout an organization, to reach busy clinicians [28]. We report here on the design, fate and impact on participants’ EBM knowledge, attitudes and skills of an EBM train-the-trainer intervention at a large university hospital aimed at reaching all of its physicians. The training rested on the assumption that many doctors had limited EBM knowledge and skills, with suboptimal ways of drawing on the scientific literature, and that a short EBM training program could influence this situation in a desirable direction.

## Methods

### Study setting

The EBM training was designed to reach all physicians at the Karolinska University Hospital, a public tertiary care academic medical center with two main campuses in Stockholm, Sweden, with approximately 15,000 employees, including 2400 salaried physicians.

### The EBM training

On the heels of a tumultuous hospital merger [29], the authors received funding to develop and offer training to all doctors in how to apply EBM in practice. In order to be able to reach all of the hospital’s 2400 doctors, we designed a two-phase “cascade” training program. Phase 1 was a train-the-trainer intervention, whereby some 100 doctors from all departments underwent training in one of six course iterations between October 2005 and March 2006 to become EBM teachers. In Phase 2, they requested materials for training their departmental colleagues.

In Phase 1, trainers attended 2 days of interactive training on the EBM process in a computer lab, where they could perform searches as the course progressed, followed by self-study over 2–3 weeks or more to apply EBM to a clinical question from their own practice and develop a Critically Appraised Topic (CAT) report [30] – a structured way to summarize evidence on a focused clinical question – modeled on BestBets (http://www.bestbets.org/) (see template in Additional file 1). The training concluded with a half day follow-up session for participants to report on their CATs and receive feedback from peers and faculty. The course covered the definition of EBM – integrating the best available scientific evidence with clinical experience and the patient’s perspective – its underlying principles and the EBM process: formulating a good question (PICO-format [31]; for Patients-Intervention-Control-Outcomes); searching for and critically appraising relevant literature; and applying findings in the clinical situation. The training included brief teaching sessions, demonstrations of relevant search engines and databases with support from hospital librarians, individual practice with faculty support, and self-directed study. The training used the Swedish edition of a course book on EBM [32], and a workbook designed by one of the authors (JN) specifically for this initiative in the hospital’s context.

**Fig. 1:**
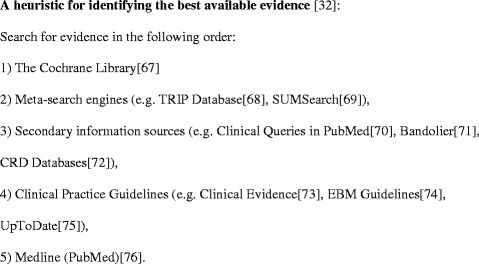
A heuristic for identifying the best available evidence. This is the order of resources recommended in the training for identifying the best available evidence [67–76]

In Phase 2, the 100 newly trained teachers were invited to request sets of the same course materials – including the course book, the workbook and the electronic template for writing a CAT – free of charge for their departmental colleagues. They also received presentation slides to explain training concepts, such as the 4-step EBM process outlined above, a heuristic for how to identify the best available evidence (see Fig. 1), critical appraisal skills, and how to communicate regarding evidence and care decisions with patients. The trainers also received a template modular curriculum (Additional file 2) which could be broken down into 45 min sessions, whereby the course content could be covered in a total of 1.5 days (12 h) at a pace tailored to each local context. The curriculum covered the same content as the trainers’ own training, including the CAT assignment, to be completed at participants’ leisure, before the concluding sessions.

Trainers were advised to solicit assistance from colleagues in their own department with special interest and skill in various aspects of the EBM process, e.g., statistics or epidemiological study design. Several trainers also enlisted hospital medical librarians to assist with training in information retrieval.

After completing their own training, trainers received a checklist for how to prepare and run the training themselves. It instructed them to contact their Department Chief to agree on when and how to teach the department’s physicians, to request course materials, to alert their colleagues about the upcoming training and ask them each to identify and bring a clinical case from their practice for use in the training, to prepare for and deliver the training, to hand out course certificates to all participants who completed a CAT, and to collect and return all questionnaires to this study’s authors.

While the training was awarded funding by the hospital management, and was meant to reach all of the hospital’s physicians, scheduling the training in the departments was in effect voluntary for department chiefs. There was no follow-up by hospital management on the dissemination of the EBM training.

### Study design and questionnaire instrument

For this observational study of the EBM training, we found the “Critical appraisal skills programme [CASP] workshop evaluation questionnaire” developed by Taylor et al. [33], geared to practicing clinicians, to be most appropriate for our purposes. This questionnaire captures demographic information (age, sex, academic training, and professional rank); current approaches to keeping up to date and to solving clinical problems; reading habits; attitudes to EBM; and, finally, knowledge of EBM assessed through 18 true-false statements. It is “a valid tool for measuring the impact of [EBM] training on participants’ knowledge, and attitudes toward [EBM].” ([33] p. 546) We translated the questionnaire into Swedish and adapted it to the local setting.

Using a before-and-after study design, we asked participants to fill in the questionnaire at the beginning of the first training session and then at the end of the concluding session, at least 2–3 weeks, and sometimes several months, later, and to use the same identifying information (Swedish social security number or cellular phone number) both times. The intention was for participants to have completed their CAT assignment at that time. The trainers received printed questionnaires to give to their colleagues, and were asked to use the same before-and-after design as they had experienced themselves.

We linked responses in pre- and post-training questionnaires at the individual level. In addition to descriptive statistics, we used Wilcoxon matched pairs test at the individual level regarding i) reading patterns (for keeping up-to-date and for solving clinical problems) and ii) responses before and after the training with respect to confidence in assessing different aspects of a published paper, approaches to solving clinical problems, attitudes to EBM, and demonstrated EBM knowledge.

For this study, we also reviewed our documentation from the planning and conduct of the training program at the hospital. This included course materials, records of course material requests, project planning documents, course evaluations and correspondence with participants and hospital managers. We drew on these materials, and our roles as faculty in the training, to describe the training program and its fate, as recommended by evaluation researchers [34].

### Research ethics

This study received ethical clearance from the Regional Board for Vetting of Research Ethics in Stockholm (#2006/33-31). Participants received written and oral information about the study and the voluntary nature of their participation. They were informed that their responses would be treated anonymously once the pre-post version linking was complete and that no information that could be linked to particular individuals would be reported. They handed in each completed questionnaire in a sealed envelope, knowing that they thereby explicitly expressed their informed consent to participate in the study.

## Results

### Reach of the training and respondent demographics

Phase 1, training-the-trainers, reached 104 doctors. The experience of these initial trainers varied; a few managed to train all their departmental colleagues, but most only reached some of their colleagues, and often only for part of the training. In several departments no training was scheduled at all, reportedly due to cost cutting pressures and staffing shortages. Ultimately, between February of 2006 and June of 2007, 60 of the 104 trained trainers requested materials for 1472 colleagues (range: 10–70 sets of materials per department), or 66 % of hospital physicians, across 42 (81 %) of the 52 departments invited to the initiative. We lack data on how many physicians actually received the materials or the training. We received 462 pre-training questionnaires (104 (100 %) from Phase 1 trainers and 358 (24 %, *n* = 1472) from their Phase 2 participants, which combined equals 19 % of the hospital’s physician population). Post-training we received 258 questionnaires (88 (85 %) and 170 (18 %), respectively, equal to 11 % of all physicians). Due to missing ID information, we were only able to connect pre- and post-training questionnaires for 174 individuals (63 (61 %) and 111 (7.5 %), equal to 7.2 % of all physicians). We analyzed questionnaire responses in this dataset, starting with respondent demographics (Table 1).

**Table 1:** Respondent demographics

Background	Phase 1 trainers (*n* = 63)	Phase 2 participants (*n* = 111)	Total (*n* = 174)
Age, mean years (standard deviation, range)	45 (8, 31–64)	44 (10, 28–67)	45 (9, 28–67)
Sex (female/male; *n*=)	27/35	59/51	86/86
	(*n* = 62)	(*n* = 110)	(*n* = 172)
Research experience:	(*n* = 63)	(*n* = 110)	(*n* = 173)
MD-PhD	56 % (*n* = 35)	48 % (*n* = 53)	51 % (*n* = 88)
MD-doctoral student	14 % (*n* = 9)	15 % (*n* = 17)	15 % (*n* = 26)
MD not in research training	30 % (*n* = 19)	36 % (*n =* 40)	34 % (*n* = 59)
Professional rank:	(*n* = 62)	(*n* = 107)	(*n* = 169)
Consultant	52 % (*n* = 32)	42 % (*n* = 45)	46 % (*n* = 77)
Specialist	31 % (*n* = 19)	21 % (*n* = 22)	24 % (*n* = 41)
Resident/intern	18 % (*n* = 11)	37 % (*n* = 40)	30 % (*n* = 51)

*Abbreviations*: *MD* Medical doctor, *PhD* Doctor of philosophy

### Information seeking behavior

#### Keeping up to date and solving clinical problems

When asked before the training “What types of resources do you use to keep up to date?”, the most common responses were *consulting a colleague* (79 % of respondents reported relying on colleagues “often/very often”), *Internet resources*, e.g., PubMed and Google (56 % of respondents reported using internet resources “often/very often”), and *textbooks* (48 % of respondents reported using textbooks “often/very often”). Less common responses were *journal articles*, e.g., original research reports and review articles (44 % of respondents reported using these “often/very often”), *electronic resources/databases available from the hospital library* (20 % of respondents reported using these “often/very often”), and *secondary journals and clinical practice guidelines*, e.g., the Cochrane Library, Clinical Evidence, and UpToDate (5 % of respondents reported using these “often/very often”).

**Fig. 2:**
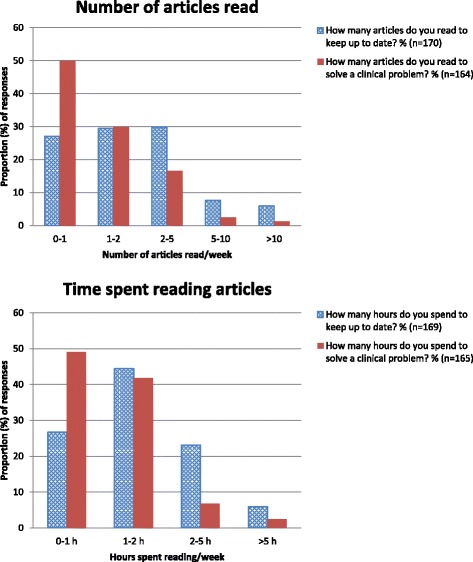
Reading to keep up to date and to solve clinical problems. The number of articles respondents reported that they read (*above*), and the time they spent reading articles (*below*), on average per week to keep up to date (*dotted blue*) and to solve a clinical problem (*solid red*)

Asked about their weekly reading patterns, respondents reported reading fewer articles (*p* < 0.0001), and spending less time (*p* < 0.0001), to solve clinical problems than to keep up to date (Fig. 2).

### Changes in reported information seeking strategies and confidence in assessment skills

The respondents’ intended use of resources to solve clinical problems changed after the training (Table 2), as did their confidence in their assessment skills (Table 3).

**Table 2:** Resources to solve a health care problem

		Total	Never/rarely	Often/very often	*p*-value*
Resources		n	%	%	
Colleagues	Before:	173	3 %	79 %	
	After:	172	2 %	65 %	0.0002
Internet resources, e.g., PubMed, Google	Before:	170	15 %	56 %	
	After:	174	1 %	85 %	<0.0001
Textbooks	Before:	173	13 %	48 %	
	After:	171	36 %	23 %	<0.0001
Journals (original research reports and review articles)	Before:	172	22 %	44 %	
	After:	171	5 %	69 %	<0.0001
Electronic resources, computer databases available from the Hospital/University library	Before:	171	64 %	20 %	
	After:	172	9 %	62 %	<0.0001
Secondary journals and Clinical Practice Guidelines, e.g., Cochrane, Clinical Evidence, EBM-Guidelines, Up To Date	Before:	170	73 %	5 %	
	After:	174	4 %	76 %	<0.0001

Distribution of responses before and after training to the survey questions ”What types of resource do you use /do you think you will use to solve a specific health care problem?” *Wilcoxon test for matched pairs – comparison of the values before and after the training

**Table 3:** Confidence in assessing studies

	Before/after	Total	Very or quite confident	Not very or not at all confident	*p-*value*
Study aspect		n	%	%	
Assessing study design	Before:	173	42 %	31 %	
	After:	173	62 %	8 %	<0.0001
Evaluating bias	Before:	172	30 %	41 %	
	After:	173	44 %	19 %	<0.0001
Evaluating the adequacy of sample size	Before:	171	22 %	51 %	
	After:	173	24 %	33 %	<0.0001
Assessing generalizability	Before:	172	30 %	41 %	
	After:	172	45 %	12 %	<0.0001
Evaluating statistical tests/principles	Before:	171	13 %	64 %	
	After:	172	19 %	42 %	<0.0001
Assessing the general worth of an article	Before:	173	45 %	25 %	
	After:	172	65 %	5 %	<0.0001

Distribution of responses before and after training to the survey question “How confident do you think that you are at assessing each of these aspects of a published paper?”

*Wilcoxon test for matched pairs – comparison variable by variable of the values before and after the training

### Impact on attitudes to EBM

Asked about their views regarding critical appraisal and the use of evidence, participants agreed particularly with three statements – even more so following the training: “by practicing EBM we increase the chances for effective care” (90 % responding “agree/agree strongly” before the training; 97 % after), “systematic reviews play a key role in informing evidence-based decision-making (from 84 to 94 %) and “study design is important in article selection” (from 80 to 92 %). Conversely, a greater proportion of respondents disagreed with the following statements after the training: “EBM has become a buzz word which does not add much to patient care” (70 % responding “disagree/disagree strongly” before the training; 91 % after it) and “evidence-based decision-making is cookbook medicine” (from 44 to 72 %). All of these changes were statistically significant (*p* = 0.024 or less).

### Changes in EBM Knowledge

The proportion of correct answers to the 18 true/false statements about the application of critical appraisal skills increased following the training, as the proportion of “Don’t know/blank” answers declined (Table 4; *p* < 0.0001).

**Table 4:** Test of critical appraisal skills

Average distribution	Incorrect answers	Don’t know/blank	Correct answers
Before training	17 %	31 %	52 %
After training	16 %	13 %	71 %

Proportion of responses before and after training to 18 true/false statements about the application of critical appraisal skills

Trainers and their participants improved equally much after the training, although trainers started at a higher proportion of correct answers before the training, (trainers went from an average of 10.1 correct answers before to 13.7 correct answers after the training while their participants went from 9.0 to 12.3 correct answers on average, both at *p* < 0.0001).

### Clinical application and participant experience

CAT reports addressed contemporary clinical problems where participants sought to compare current practice with the best available evidence. They included, for example, assessment of whether to operate or practice watchful waiting for patients with peptic ulcer perforation; whether anticoagulation treatment affects the pregnancy outcome among women with multiple spontaneous abortions of unknown cause; and whether local injection with a corticosteroid would prevent recurrence of keloid formation on the ear. Participants indicated that they would change their practice in light of their CAT findings and, in some cases, that their CAT had prompted updating of departmental clinical guidelines.

Participants’ course evaluations were predominantly favorable regarding both the content and educational design. Free-text comments (translated from Swedish) included:

*“I’ve gained a structured approach and thereby a lower threshold for applying EBM.”*

*“[The training introduced] important search engines to get answers to clinical questions.”*

*“EBM isn’t so scary and difficult – you have defused it very well.”*

*“I ought to have had this type of training at the beginning of my career.”*

## Discussion

This study supports the initial assumptions that participating doctors had limited EBM knowledge and skills, with suboptimal ways of drawing on the scientific literature, and that a short EBM training program could influence this situation in a desirable direction. Before the training, participants reported that they most commonly relied on colleagues to solve clinical problems, mirroring studies elsewhere [35, 36]. This is problematic [37] since colleagues’ opinions may not reflect the best available evidence. Respondents’ second most common pre-training resource was Internet search engines (PubMed, Google) while only a few used readily available secondary sources of evidence, such as the Cochrane Library, generally recognized as providing the best evidence in a usable and understandable format [38]. The training instead promoted another priority for information seeking (see Fig. 1). This priority is reflected in post-training responses regarding which sources participants anticipated using to solve clinical problems after the training, e.g., the propensity to use secondary sources of evidence, which rose from 5 to 76 % – a large and welcome shift [28].

Participants reported reading more articles and spending more time keeping up to date than solving clinical problems. This can be characterized as learning “just-in-case” to prepare for potential future clinical problems – rather than drawing on the literature in a more focused manner guided by actual current patient needs – learning “just-in-time” [27, 39]. Just-in-time learning involves the use of PICO elements, integral to the EBM process [31]. Cheng [40] and Green et al. [41]. have found that the more senior the professional, the more likely she or he is to use just-in-time learning. Most respondents in this study were clearly senior (more than 50 % held MD-PhD degrees) and it is unclear why our results are at odds with previous studies. The training promoted just-in-time learning as a way to manage the “information overload” caused by the ever-increasing number of scientific publications in medicine. No one can keep abreast of all relevant developments concerning their field of practice by reading new publications “just-in-case”; certainly not if reading on average 1–2 articles per week.

Participants’ confidence in appraising scientific articles – particularly study design, bias, generalizability, and the general worth of an article – increased following the training. Furthermore, a greater proportion of respondents agreed that EBM increases the chances for effective care and disagreed with the claim that EBM is “cookbook medicine”. Contrary to earlier research [21], this supports the idea that doctors’ attitudes and skills can be influenced, even by a short training program [23, 42, 43]. We surmise that the more favorable clinicians’ attitudes to EBM are, the greater will be their propensity to actually practice EBM, although this has proven difficult to demonstrate empirically [43, 44]. Prior to the training, the proportion of correct answers to the true/false statements designed to gauge respondents’ critical appraisal skills was similar to what could be achieved through random guesswork. After the course, the proportion of correct answers increased from 52 to 71 % and “don’t know” responses decreased (from 31 to 13 %). The impact was similar among trainers and their phase 2 trainees, suggesting the potential effectiveness of using a train-the-trainer approach [45]. The changes associated with this short training were substantial and encouraging, even if there was room for further improvement, here as in other settings [22, 25, 42, 46].

The curricular design in earlier EBM-studies has often been poorly described [21] which is unfortunate, since it is essential for understanding the mechanisms of training effectiveness [34, 47]. The design reported here, while involving classroom sessions, exhibits most features of the highest level in a research-based hierarchy of effective EBM teaching and learning methods [27]. We hypothesize that the learner-centered, patient-related, participant-activating, problem-based design – with modules that could be delivered in 45 min installments – and the concluding CAT assignment, contributed to the impact of the training. An international survey of EBM teachers identified language as a barrier to learning in non-Anglophone countries [48]. In our study setting, although proficiency in English is widespread, providing instruction and teaching materials in Swedish might have promoted the training’s impact.

While we learned that participants’ CAT reports directly led to changes in local practice and to departmental guidelines in a few cases – indicating that the training was clinically relevant – further research is needed to unpack the effectiveness of this and other curricular designs, including e-learning formats [23, 24, 49–51]. While many e-learning designs lack room for interactivity and higher-level thinking [52], the present design including the CAT assignment, provided such opportunities. This should be considered in the on-going debate about effective educational designs [24].

The training, though relatively short, did not reach as far as intended, despite the centrality of EBM to the mission of a university hospital. We have no indications that the fate of the training, i.e., to not conduct it at all or only partially in some departments, was due to a lack of interest in EBM as such on the part of clinicians and managers, or that EBM knowledge was already considered sufficient there. It is possible that the Phase 1 training was too limited to give all trainers enough confidence in their ability to replicate it in Phase 2 in their own departments. Since participants gave very favorable feedback on the training, however, we have no reason to believe that the training itself discouraged spread. Instead, competing demands seem to have hampered the full dissemination of the training, possibly related to post-merger cost cutting and re-organization [29], mirroring challenges found in similar situations elsewhere [53, 54]. The hospital leadership, after funding the training, did not ensure that it reached all hospital physicians; some department chiefs reported that while they supported the training in principle, they were unable under the circumstances to make room for it. The limited reach of the EBM training thus illustrates how leadership support is essential for organizational improvement initiatives [55] and how its absence reduces the likelihood of success [56].

### Study limitations and methodological considerations

Firstly, we received completed questionnaires only from a minority of potential participants at the hospital. While course materials were distributed for over 1500 doctors, we only received 462 pre-training and 258 post-training questionnaires; the training clearly failed to reach the entire intended audience. We do not fully know why. In phase 2, the training was the responsibility of the trainers and their departmental chiefs and colleagues. The limited reach of the training signals a gap between the intention – as initially proposed to, and endorsed by, hospital leadership – and the ability/propensity/willingness among departmental chiefs and physicians to actually carry out the training, as indicated above.

While the training thus had a limited reach, we believe it is still important to report on the data we were able to collect, both because they indicate important possibilities to enhance EBM practices and because the challenges in reaching all intended participants and in data collection can inform future interventions and studies [34]. The questionnaire responses should be generalized to the entire population of physicians at the study hospital (or beyond) with caution since we cannot determine how representative the respondents are. That said, they do show that it is possible to influence university hospital physicians’ EBM attitudes and knowledge in a desirable way with this type of short training program, and that a train-the-trainer design *can extend* this influence also to a phase 2. If we can assume that participants were more, rather than less, positive towards EBM than the entire population of physicians, the findings are both disconcerting and encouraging. They are disconcerting because at baseline, EBM knowledge and practice left much to be desired. They are encouraging because it was possible, even with such a short training, to achieve a significant change for the better.

The final dataset included 174 respondents for whom we were able to connect questionnaires before and after the training (a strategy employed also in a cluster randomized controlled trial comparing two EBM training modalities [46]). Rather than performing analyses using all questionnaires at group level (yielding a larger study population), we chose to restrict our analyses to this dataset with before-and-after responses linked for each individual to gain greater confidence when assessing the direction and size of changes. Respondents were asked to identify themselves on both the pre- and post-training questionnaire; meant to be cost-effective, this turned out to be an ineffective design. A similar challenge in capturing respondent identity occurred in a German setting [18]. Better ways to link repeated questionnaires at an individual level, while protecting respondents’ integrity, are clearly needed.

Secondly, the limited scope of the evaluation using the validated questionnaire [33], with its restricted focus on reported reading patterns, critical appraisal, knowledge, skills and attitudes regarding EBM, did not enable us to assess changes in participants’ *actual* practice patterns or related patient outcomes. Intended future practice patterns following the training may not be borne out as reported. Respondents may have been susceptible to social desirability, thus projecting overly favorable practice changes [57]. The fact that the questionnaire was self-administered and that responses were treated confidentially should limit this tendency [58]. The challenge of linking EBM training to changes in clinical practice and patient outcomes is not unique for this study but remains a worthy goal for methodological development [20, 23, 25, 51, 59–61]. A rare example of such linking showed that an EBM-intervention led to more evidence-based practice [19]. A systematic review of 113 EBM training assessment studies found that only 10 % (*n* = 11) measured change in participant behavior; only 3 % (*n* = 4) measured changes in the delivery of care [62]. The authors deemed it “surprising that so few teaching efforts were accompanied by appropriately developed assessments.” Related to concerns about our training’s impact on practice are concerns about its durability. While not possible for us (and rare in reviews of studies [23]), it would have been interesting to follow respondents over time, to assess whether EBM skills and attitudes persisted and developed or dwindled over time.

Thirdly, a before-and-after study design carries inherent potential weaknesses, particularly regarding the potential confounding effect of a secular trend in EBM knowledge. The use of a control group, and random assignment of study participants to either training or control group, while not feasible in our situation, could have strengthened our confidence in attributing the changes in participants’ reading patterns, knowledge and attitudes, to the EBM training and has subsequently been employed elsewhere [62–64]. We have no particular reason, however, to believe that any secular trend or other concurrent initiatives confounded the effects of the training captured by questionnaires before and after the training. Using a similar design to evaluate an e-EBM training program, Kulier et al. noted that “the assessments before the course served as control for each individual” and that “we can be reasonably sure the gain in knowledge was due to” the training [42].

Given that the questionnaires were similar before and after the training, respondents might remember some of the questions (e.g., true/false statements) from before the training and then identify the correct answer before completing the after-training questionnaire. If so, the before-training survey played a kind of diagnostic role, by alerting respondents to areas where they needed to gain more knowledge. Any learning effects then arguably were real, in the sense that participants had to know the subject matter to get the answers right. We did not go over, or hand out, the correct answers to the true/false statements at any point – memorizing questionnaire item responses by heart thus was not possible. Therefore, we suggest that the changes in responses are attributable to learning from the training.

### Implications for practice and further research

Our findings suggest that not all clinicians – in this case at an academic medical center – possessed basic EBM knowledge and skills nor used the scientific literature optimally. This can help explain the observed gaps between the best available evidence and clinical practice [3–5]. Given that some physicians have not had any EBM training, and that the short training we designed and report on here had a favorable impact on EBM knowledge, attitudes and skills, we suggest that this kind of training can be beneficial and should be offered widely. It remains an important research task to assess the impact on clinical practice and patient outcomes of such training initiatives, as well as to develop and evaluate educational designs and clinical support systems that enable clinicians to learn about EBM and integrate it into their daily practice within existing resource constraints [65, 66].

## Conclusions

Given how central EBM is to the mission of doctors and healthcare organizations, there is room for substantial improvement regarding its application in practice. The short EBM training evaluated in this study promoted knowledge, skills and attitudes conducive to such application among the minority of hospital doctors who were able to participate. If similar training were offered and supported widely, with concurrent evaluative studies, it could help improve clinical practice and patient health while developing the knowledge base for doing so.

## Additional files

Additional file 1: The Critically Appraised Topic template. (DOCX 16 kb) Additional file 2: Template modular EBM curriculum. (DOCX 19 kb)

## Abbreviations

CAT: Critically appraised topic
EBM: Evidence-based medicine
ID: Identity
MD: Medical doctor
PhD: Doctor of philosophy
PICO: Patients-intervention-control-outcomes

## References

[1] Sackett DL, Rosenberg WMC, Gray JAM, Haynes RB, Richardson WS (1996). Evidence based medicine: what it is and what it isn’t. BMJ.

[2] Davidoff F, Haynes B, Sackett D, Smith R (1995). Evidence based medicine. BMJ.

[3] Institute of Medicine (U.S.); Committee on Quality of Health Care in America (2001). Crossing the quality chasm : a new health system for the 21st century.

[4] McGlynn EA, Asch SM, Adams J, Keesey J, Hicks J, DeCristofaro A, Kerr EA. The quality of health care delivered to adults in the United States. N Engl J Med. 2003;348(26):2635–45. 10.1056/NEJMsa022615 12826639

[5] Stenestrand U, Lindback J, Wallentin L (2005). Hospital therapy traditions influence long-term survival in patients with acute myocardial infarction. Am Heart J.

[6] Davies K, Harrison J (2007). The information-seeking behaviour of doctors: a review of the evidence. Health Info Libr J.

[7] van Dijk N, Hooft L, Wieringa-de Waard M (2010). What are the barriers to residents’ practicing evidence-based medicine? A systematic review. Acad. Med.

[8] WMA Declaration on Guidelines for Continuous Quality Improvement In Health Care [http://www.wma.net/en/30publications/10policies/g10/index.html]

[9] The WHO agenda [http://www.who.int/about/agenda/en/index.html]

[10] Institute of Medicine (2008). Evidence-based medicine and the changing nature of health care: 2007 IOM annual meeting summary.

[11] Kunz R, Nagy E, Coppus SF, Emparanza JI, Hadley J, Kulier R, Weinbrenner S, Arvanitis TN, Burls A, Cabello JB et al. How far did we get? How far to go? A European survey on postgraduate courses in evidence-based medicine. J Eval Clin Pract. 2009;15(6):1196–204. 10.1111/j.1365-2753.2009.01268.x 20367727

[12] Bednarczyk J, Pauls M, Fridfinnson J, Weldon E (2014). Characteristics of evidence-based medicine training in Royal College of Physicians and Surgeons of Canada emergency medicine residencies - a national survey of program directors. BMC Med Educ.

[13] Prasad K (2012). Teaching evidence-based medicine in resource-limited countries. JAMA.

[14] Choudhry NK, Fletcher RH, Soumerai SB (2005). Systematic review: the relationship between clinical experience and quality of health care. Ann Intern Med.

[15] Evidence-Based Medicine Working Group, Guyatt G, Cairns J, Churchill D, Cook D, Haynes B, Hirsh J, Irvine J, Levine M, Levine M et al. Evidence-based medicine. JAMA. 1992;268(17):2420–5. 10.1001/jama.1992.03490170092032 1404801

[16] Coomarasamy A, Khan KS (2004). What is the evidence that postgraduate teaching in evidence based medicine changes anything? A systematic review. BMJ.

[17] Leipzig RM, Wallace EZ, Smith LG, Sullivant J, Dunn K, McGinn T (2003). Teaching evidence-based medicine: a regional dissemination model. Teach Learn Med.

[18] Fritsche L, Greenhalgh T, Falck-Ytter Y, Neumayer H-H, Kunz R (2002). Do short courses in evidence based medicine improve knowledge and skills? Validation of Berlin questionnaire and before and after study of courses in evidence based medicine. BMJ.

[19] Straus SE, Ball C, Balcombe N, Sheldon J, McAlister FA (2005). Teaching evidence-based medicine skills can change practice in a community hospital. J Gen Intern Med.

[20] Hatala R, Guyatt G (2002). Evaluating the teaching of evidence-based medicine. JAMA.

[21] Coomarasamy A, Taylor R, Khan KS (2003). A systematic review of postgraduate teaching in evidence-based medicine and critical appraisal. Med Teach.

[22] Flores-Mateo G, Argimon JM (2007). Evidence based practice in postgraduate healthcare education: a systematic review. BMC Health Serv Res.

[23] Young T, Rohwer A, Volmink J, Clarke M (2014). What are the effects of teaching evidence-based health care (EBHC)? Overview of systematic reviews. PLoS One.

[24] Phillips AC, Lewis LK, McEvoy MP, Galipeau J, Glasziou P, Hammick M, Moher D, Tilson JK, Williams MT:. A systematic review of how studies describe educational interventions for evidence-based practice: stage 1 of the development of a reporting guideline. BMC Med Educ. 2014;14:152. 10.1186/1472-6920-14-152 PMC4113129 25060160

[25] Kunz R, Wegscheider K, Fritsche L, Schunemann HJ, Moyer V, Miller D, Boluyt N, Falck-Ytter Y, Griffiths P, Bucher HC et al. Determinants of knowledge gain in evidence-based medicine short courses: an international assessment. Open med. 2010;4(1):e3–e10. 10.2174/1874104501004010003 PMC3116678 21686291

[26] Green ML, Ellis PJ (1997). Impact of an evidence-based medicine curriculum based on adult learning theory. J Gen Intern Med.

[27] Khan KS, Coomarasamy A (2006). A hierarchy of effective teaching and learning to acquire competence in evidenced-based medicine. BMC Med Educ.

[28] Carpenter CR, Kane BG, Carter M, Lucas R, Wilbur LG, Graffeo CS (2010). Incorporating evidence-based medicine into resident education: a CORD survey of faculty and resident expectations. Acad. Emerg. Med.

[29] Choi S (2011). Competing logics in hospital mergers - The case of the Karolinska University Hospital.

[30] Sauvé S, Lee HN, Meade MO, Lung JD, Farkouh M, Cook DJ, Sackett DL. The critically appraised topic: a practical approach to learning critical appraisal. Ann R Coll Physicians Surg Can. 1995;28(7):3.

[31] Sackett DL (2000). Evidence-based medicine : how to practice and teach EBM.

[32] Nordenström J (2007). Evidence-based medicine in Sherlock Holmes’ footsteps.

[33] Taylor R, Reeves B, Mears R, Keast J, Binns S, Ewings P, Khan K. Development and validation of a questionnaire to evaluate the effectiveness of evidence-based practice teaching. Med Educ. 2001;35(6):544–7. 10.1046/j.1365-2923.2001.00916.x 11380856

[34] Olson CA, Bakken LL (2013). Evaluations of educational interventions: getting them published and increasing their impact. J Contin Educ Heal Prof.

[35] Barrie AR, Ward AM (1997). Questioning behaviour in general practice: a pragmatic study. BMJ.

[36] McAlister FA, Graham I, Karr GW, Laupacis A (1999). Evidence-based medicine and the practicing clinician. J Gen Intern Med.

[37] Schaafsma F, Verbeek J, Hulshof C, van Dijk F (2005). Caution required when relying on a colleague’s advice; a comparison between professional advice and evidence from the literature. BMC Health Serv Res.

[38] Grandage KK, Slawson DC, Shaughnessy AF (2002). When less is more: a practical approach to searching for evidence-based answers. J Med Libr Assoc.

[39] Coppus SF, Emparanza JI, Hadley J, Kulier R, Weinbrenner S, Arvanitis TN, Burls A, Cabello JB, Decsi T, Horvath AR et al. A clinically integrated curriculum in evidence-based medicine for just-in-time learning through on-the-job training: the EU-EBM project. BMC Med Educ. 2007;7:46. 10.1186/1472-6920-7-46 PMC2228282 18042271

[40] Cheng GY (2004). A study of clinical questions posed by hospital clinicians. J Med Libr Assoc.

[41] Green ML, Ciampi MA, Ellis PJ (2000). Residents’ medical information needs in clinic: are they being met?. Am. J. Med.

[42] Kulier R, Hadley J, Weinbrenner S, Meyerrose B, Decsi T, Horvath AR, Nagy E, Emparanza JI, Coppus SF, Arvanitis TN et al. Harmonising evidence-based medicine teaching: a study of the outcomes of e-learning in five European countries. BMC Med Educ. 2008;8:27. 10.1186/1472-6920-8-27 PMC2386125 18442424

[43] Shuval K, Shachak A, Linn S, Brezis M, Feder-Bubis P, Reis S (2007). The impact of an evidence-based medicine educational intervention on primary care physicians: a qualitative study. J Gen Intern Med.

[44] Shuval K, Berkovits E, Netzer D, Hekselman I, Linn S, Brezis M, Reis S. Evaluating the impact of an evidence-based medicine educational intervention on primary care doctors’ attitudes, knowledge and clinical behaviour: a controlled trial and before and after study. J Eval Clin Pract. 2007;13(4):581–98. 10.1111/j.1365-2753.2007.00859.x 17683300

[45] der Leeuw HGAR J-v, van Dijk N, de Jong W, Wieringa-de Waard M (2014). Educating the clinical trainer: professional gain for the trainee? A controlled intervention study in general practice. Perspect Med Educ.

[46] Hadley J, Kulier R, Zamora J, Coppus SF, Weinbrenner S, Meyerrose B, Decsi T, Horvath AR, Nagy E, Emparanza JI et al. Effectiveness of an e-learning course in evidence-based medicine for foundation (internship) training. J R Soc Med. 2010;103(7):288–94. 10.1258/jrsm.2010.100036 PMC2895523 20522698

[47] Glasziou P (2006). What is EBM and how should we teach it?. Med Teach.

[48] Oude Rengerink K, Thangaratinam S, Barnfield G, Suter K, Horvath AR, Walczak J, Welminska A, Weinbrenner S, Meyerrose B, Arvanitis TN et al. How can we teach EBM in clinical practice? An analysis of barriers to implementation of on-the-job EBM teaching and learning. Med Teach. 2011;33(3):e125–130. 10.3109/0142159X.2011.542520 21345051

[49] Thangaratinam S, Barnfield G, Weinbrenner S, Meyerrose B, Arvanitis TN, Horvath AR, Zanrei G, Kunz R, Suter K, Walczak J et al. Teaching trainers to incorporate evidence-based medicine (EBM) teaching in clinical practice: the EU-EBM project. BMC Med Educ. 2009;9:59. 10.1186/1472-6920-9-59 PMC2753626 19744327

[50] Weberschock T, Sorinola O, Thangaratinam S, Oude Rengerink K, Arvanitis TN, Khan KS, Group EBMU. How to confidently teach EBM on foot: development and evaluation of a web-based e-learning course. Evid Based Med. 2013;18(5):170–2. 10.1136/eb-2012-100801 22864372

[51] Leung EY, Malick SM, Khan KS, Collaboration E-C (2013). On-the-Job evidence-based medicine training for clinician-scientists of the next generation. Clin. Biochem. Rev.

[52] Foster MJ, Shurtz S, Pepper C (2014). Evaluation of best practices in the design of online evidence-based practice instructional modules. J Med Libr Assoc.

[53] Fulop N, Protopsaltis G, Hutchings A, King A, Allen P, Normand C, Walters R. Process and impact of mergers of NHS trusts: multicentre case study and management cost analysis. BMJ. 2002;325(7358):246. 10.1136/bmj.325.7358.246 PMC117637 12153920

[54] Humphries S, Stafinski T, Mumtaz Z, Menon D (2014). Barriers and facilitators to evidence-use in program management: a systematic review of the literature. BMC Health Serv Res.

[55] Batalden P, Splaine M (2002). What will it take to lead the continual improvement and innovation of health care in the twenty-first century?. Qual. Manag. Health Care.

[56] Ovretveit J (2005). Leading improvement. J Health Organ Manag.

[57] van de Mortel TF (2008). Faking it: social desirability response bias in self-report research. Aust J Adv Nurs.

[58] Tourangeau R, Smith TW (1996). Asking sensitive questions: the impact of data collection mode, question format, and question context. Public Opin. Q.

[59] Shaneyfelt T, Baum KD, Bell D, Feldstein D, Houston TK, Kaatz S, Whelan C, Green M. Instruments for evaluating education in evidence-based practice: a systematic review. JAMA. 2006;296(9):1116–27. 10.1001/jama.296.9.1116 16954491

[60] Tilson JK, Kaplan SL, Harris JL, Hutchinson A, Ilic D, Niederman R, Potomkova J, Zwolsman SE. Sicily statement on classification and development of evidence-based practice learning assessment tools. BMC Med Educ. 2011;11:78. 10.1186/1472-6920-11-78 PMC3221624 21970731

[61] Walczak J, Kaleta A, Gabrys E, Kloc K, Thangaratinam S, Barnfield G, Weinbrenner S, Meyerrose B, Arvanitis TN, Horvath AR et al. How are “teaching the teachers” courses in evidence based medicine evaluated? A systematic review. BMC Med Educ. 2010;10:64. 10.1186/1472-6920-10-64 PMC2958160 20920240

[62] Malick SM, Hadley J, Davis J, Khan KS (2010). Is evidence-based medicine teaching and learning directed at improving practice?. J R Soc Med.

[63] Kulier R, Khan KS, Gulmezoglu AM, Carroli G, Cecatti JG, Germar MJ, Lumbiganon P, Mittal S, Pattinson R, Wolomby-Molondo JJ et al. A cluster randomized controlled trial to evaluate the effectiveness of the clinically integrated RHL evidence -based medicine course. Reprod Health. 2010;7:8. 10.1186/1742-4755-7-8 PMC2880979 20470382

[64] Kulier R, Gulmezoglu AM, Zamora J, Plana MN, Carroli G, Cecatti JG, Germar MJ, Pisake L, Mittal S, Pattinson R et al. Effectiveness of a clinically integrated e-learning course in evidence-based medicine for reproductive health training: a randomized trial. JAMA. 2012;308(21):2218–25. 10.1001/jama.2012.33640 23212499

[65] Hayes CW, Batalden PB, Goldmann D (2015). A ‘work smarter, not harder’ approach to improving healthcare quality. BMJ Qual Saf.

[66] Glasziou P, Ogrinc G, Goodman S (2011). Can evidence-based medicine and clinical quality improvement learn from each other?. BMJ Qual Saf.

[67] The Cochrane Library [http://www.cochranelibrary.com/]

[68] TRIP [http://www.tripdatabase.com/]

[69] SUMSearch 2 [http://sumsearch.org/]

[70] PubMed Clinical Queries [http://www.ncbi.nlm.nih.gov/pubmed/clinical]

[71] Bandolier [www.medicine.ox.ac.uk/bandolier]

[72] The CRD Database [http://www.crd.york.ac.uk/CRDWeb/]

[73] Clinical Evidence [http://clinicalevidence.bmj.com/x/index.html]

[74] Evidence-Based Medicine Guidelines [http://www.ebm-guidelines.com/dtk/ebmg/home]

[75] UpToDate [http://www.uptodate.com/home]

[76] PubMed [http://www.ncbi.nlm.nih.gov/pubmed]

