# Supplementary material for: The design, fate and impact of a hospital-wide training program in evidence-based medicine for physicians – an observational study
Source: BMC Med Educ. 2016 Mar 8;16:86. doi: 10.1186/s12909-016-0601-9 (PMC4784409; doi:10.1186/s12909-016-0601-9)
Supplement: Additional file 1: — The Critically Appraised Topic template. (DOCX 16 kb) [file 12909_2016_601_MOESM1_ESM.docx]

**Instruction:** Fill out the sections below according to the directions.

**Title:** (Let the title summarize your conclusion)

**Author:** (your name)

**Department:** (your department)

**Date:** (date of completion)

**Clinical question:** (PICO structure), e.g. ”In [an adult for whom a foreign body has been removed from the cornea] do [topical antibiotics] reduce [the time until healing]?”

**Clinical scenario:** (describe a real or made-up case)

**Search strategy:** (describe which data bases you have searched and note which search terms [MeSH or free text words] you have used and which Boolean search terms you have used [AND, OR, NOT]).

**Search results:** (the number of articles on the topic and what number of them were relevant to your clinical question)

**Relevant articles:** (preferably in table format like in BestBets with: 1) The first author’s name and the year of publication. 2) Patient groups. 3) Type of study design. 4) Outcome measures used. 5) Results. 6) Potential weaknesses.).

| **1) Author, year** | **2) Patient groups** | **3) Design** | **4) Outcome measures** | **5) Results** | **6) Potential weaknesses** |
| --- | --- | --- | --- | --- | --- |
|  |  |  |  |  |  |
|  |  |  |  |  |  |
|  |  |  |  |  |  |

**Comments, if any:**

**Evidence strength grading:** (A, B, C, or D)

**Conclusion:**

**References:** ([First author, title, journal, volume, pages, year] for the articles you commented on above)
