# Supplementary material for: The design, fate and impact of a hospital-wide training program in evidence-based medicine for physicians – an observational study
Source: BMC Med Educ. 2016 Mar 8;16:86. doi: 10.1186/s12909-016-0601-9 (PMC4784409; doi:10.1186/s12909-016-0601-9)
Supplement: Additional file 2: — Template modular EBM curriculum. (DOCX 19 kb) [file 12909_2016_601_MOESM2_ESM.docx]

| **Time** |  | **Day 1** |  | **Departmental meeting 1** |  | | **Departmental meeting 2, 3 etc.** |
| --- | --- | --- | --- | --- | --- | --- | --- |
| 08.30-09.15 |  | **Welcome**  **Questionnaire I (*before* training) administered**  **Introduction to the course**   - **The work book, p. 4-5**   **What is EBM?**   - **Reading 10 min. Sherlock p. 7-12** - **The work book, p. 6-8** |  | **Step 3 (cont.)**  **Discussion of a selected article.**  **The work book, p. 16-19** |  | | **Examination: report of individual study.**  **(5-6 reports/hour)**  **Evaluation, questionnaire II (*after* training). Course certificate given to all who have reported their invididual CAT assignments.** |
| 09.15-10.00 |  | **Step 1 = Formulating a question**   - **Reading 15 min. Sherlock p. 13-15** - **The work book, p. 9** |  | **Step 4 = Application.**   - **Reading 10 min., Sherlock p. 93-99** - **The work book, p. 25** |  | |  |
| BREAK |  |  |  |  |  | |  |
| 10.30-11.15 |  | **Participants design their own case presentation using PICO.**  **Presentation of the cases.** |  |  |  | |  |
| 11.15-12.00 |  | **Information sources for EBM.**   - **Reading 20 min. Sherlock p. 16-34** - **The work book, p. 10-11 + 30-36** |  |  |  | |  |
| LUNCH |  |  |  |  |  | |  |
| 13.00-13.45 |  | **Information sources (cont.)** |  |  |  | | |
| 13.45-14.30 |  | **Step 2 = Information searching.**   - **Reading 30 min., Sherlock p. 35-52**   **The work book, p. 12-13 + 37-39** |  |  |  |  | |
| BREAK |  |  |  |  |  |  | |
| 15.00-15.45 |  | **Step 2 (cont.)** |  |  |  | | |
| 15.45-16.30 |  | **Step 3 = Critical appraisal, incl. therapy studies, diagnostic tests, systematic reviews/ meta-analyses, clinical guidelines.**   - **Read 30 min., Sherlock p. 53-92** - **The work book, p. 14-23** |  |  |  |  | |
